# A Review of Machine Learning Approaches for the Personalization of Amplification in Hearing Aids

**DOI:** 10.3390/s24051546

**Published:** 2024-02-28

**Authors:** Nafisa Zarrin Tasnim, Aoxin Ni, Edward Lobarinas, Nasser Kehtarnavaz

**Affiliations:** 1 Electrical and Computer Engineering Department, University of Texas at Dallas, Richardson, TX 75080, USA; nafisazarrin.tasnim@utdallas.edu (N.Z.T.); aoxin.ni@utdallas.edu (A.N.); 2 Callier Center for Communication Disorders, University of Texas at Dallas, Richardson, TX 75080, USA; edward.lobarinas@utdallas.edu

**Keywords:** personalization of amplification in hearing aids, machine learning approaches to the personalization of hearing aids, personalized hearing aid fitting

## Abstract

This paper provides a review of various machine learning approaches that have appeared in the literature aimed at individualizing or personalizing the amplification settings of hearing aids. After stating the limitations associated with the current one-size-fits-all settings of hearing aid prescriptions, a spectrum of studies in engineering and hearing science are discussed. These studies involve making adjustments to prescriptive values in order to enable preferred and individualized settings for a hearing aid user in an audio environment of interest to that user. This review gathers, in one place, a comprehensive collection of works that have been conducted thus far with respect to achieving the personalization or individualization of the amplification function of hearing aids. Furthermore, it underscores the impact that machine learning can have on enabling an improved and personalized hearing experience for hearing aid users. This paper concludes by stating the challenges and future research directions in this area.

## 1. Introduction

Hearing impairment poses a significant challenge for individuals, affecting not only their ability to communicate effectively but also impacting their overall quality of life. Despite the widespread use of hearing aids, nearly one half of hearing aid users report not being satisfied with their hearing aid fittings [1]. A major reason for this lack of satisfaction stems from the generic aspect of these fittings, which do not fully incorporate an individual user’s hearing preferences in audio environments in which the user finds it challenging to communicate. The consequences of suboptimal hearing aid fittings extend beyond communication difficulties and can result in overall reductions in overall quality of life [2,3]. These consequences are particularly pronounced among the elderly, for whom hearing loss is not only prevalent but also correlated with a higher risk of developing or exacerbating dementia [4].

This paper provides a review of published articles that use machine learning approaches for the personalization of the amplification function of hearing aids. These approaches employ various machine learning methodologies, including hierarchical preference learning, expectation–maximization, linear regression, Bayesian learning, neural networks, transfer learning, deep reinforcement learning, maximum likelihood inverse reinforcement learning, Gaussian processes, dueling multi-armed bandit, and active learning. The use of these machine learning methods for personalizing the amplification function of hearing aids has enabled the enhancement of the hearing experience of their users. In addition, this review also includes articles that do not explicitly mention machine learning but discuss the importance of personalization in hearing aid healthcare as analyzed in studies conducted either in a laboratory or real-world audio environments.

The focus of this paper is on the approaches that address user preferences in different audio environments. This review examines the current landscape of machine learning approaches for hearing aid personalization and the promising strategies that can potentially alter the way the hearing aid fitting process is currently conducted.

## 2. Overview of Hearing Aids and Their Amplification Function

**Figure 1:**
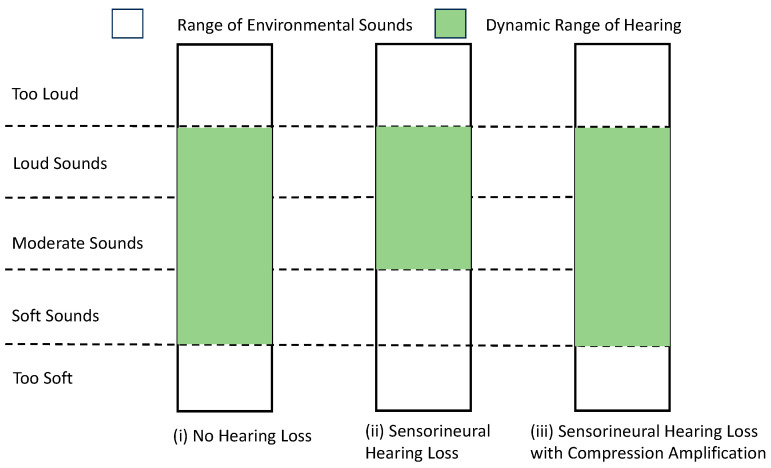
Dynamic range of hearing: (i) no hearing loss, (ii) sensorineural hearing loss, and (iii) sensorineural hearing loss with amplification achieved via compression.

Sensorineural hearing loss is characterized by specific auditory challenges, such as increased difficulty in perceiving low-intensity, high-frequency speech sounds and hearing in an environment with competing noise [5]. Mild hearing loss is typically characterized by difficulty hearing softer sounds, especially with background noise, whereas profound hearing loss may result in an inability to hear even loud sounds regardless of whether background noise is present. Figure 1 depicts the dynamic range of hearing of a person with (i) no hearing loss, (ii) sensorineural hearing loss, and (iii) sensorineural hearing loss with amplification achieved via compression. As illustrated in Figure 1, the reduction in dynamic range presents a major problem because soft and intermediate sounds may be inaudible but loud sounds are still perceived as loud. The net result is that the range between barely audible and intolerably loud is significantly reduced. As such, there is a reduction in the operating space in which to fit sounds that range from soft to loud. Moreover, hearing-impaired individuals differ in their preferences as to where the optimal amplification setting is within the residual dynamic range.

**Figure 2:**
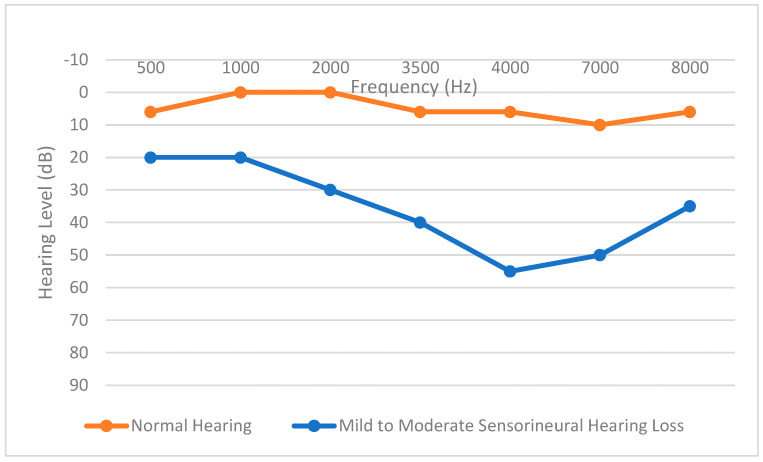
Audiogram for a normal hearing case and for a hearing loss case.

Currently, the prescriptive approaches are largely based on population data and rely on auditory thresholds assessed using an audiogram and the average or individual loudness discomfort level. These two values set the dynamic range. With respect to thresholds, an audiogram, a graphical representation of the softest sounds (threshold) a person can hear at different frequencies, plays a crucial role in documenting, assessing, and categorizing hearing loss [6,7]. The horizontal axis of an audiogram represents a subset of sound frequencies audible to humans, ranging from low to high frequencies measured in Hertz (Hz), and the vertical axis represents the intensity or loudness of sounds measured in decibels (dB). An audiogram is a visual representation of an individual’s hearing thresholds in different frequency bands essential for speech comprehension. Figure 2 shows typical audiograms for a person considered to have hearing within normal limits (top curve) and for an individual with mild to moderate sensorineural hearing loss (bottom curve).

Hearing aids are the primary and most effective interventions used to address the needs of individuals with hearing loss and work by amplifying sounds that are inaudible or barely audible in order to enhance hearing and thus allow speech comprehension [8]. Hearing aids are normally prescribed by a hearing healthcare professional. However, more recently, the US Food and Drug Administration (FDA) approved over-the-counter (OTC) hearing aids. Traditional prescribed hearing aids involve a standardized fitting process performed by a hearing care professional aimed at providing audibility based on the normative and well-documented amplification needs of individuals with similar forms of hearing loss.

Aggregate data from individuals with hearing loss have led to a number of hearing aid prescriptions, some of which are universal, whereas others are manufacturer-specific [9]. Examples of widely used universal prescriptions include NAL-NL1 [10], NAL-NL2 [11,12], and DSL [13,14,15]. Another widely used prescription that is currently available is Adaptive Dynamic Range Optimization (ADRO). A differentiating feature of ADRO is that it adjusts the amplification level in an adaptive manner [16,17,18,19]. When fitting hearing aids, the overall goal of the prescriptive approach is to make soft sounds audible, moderate sounds comfortable, and loud sounds tolerable in order to maintain speech at a comfortable and optimal listening level. Amplification is generally achieved through the use of sound compression across frequencies, which allows adapting the world of sound such that it can be compressed into the smaller dynamic range of hearing of individuals with hearing loss [20,21,22]. This is illustrated in Figure 1. Hearing care professionals fine-tune settings to cater to individual needs due to the diversity of user preferences and audio environments. However, there is no widely accepted or standardized method for making these fine adjustments.

The pilot study outlined in [23] demonstrated individual preferences by allowing users to choose from various settings while listening to Bluetooth-connected hearing aids. The cited study assessed individual preferences related to program and volume adjustments. The participants exhibited different ways of adjusting volume, with some preferring program changes, whereas others actively used volume adjustments to fine-tune their experiences. The findings showed significant variability in user behavior, reinforcing the need for personalized settings.

**Figure 3:**
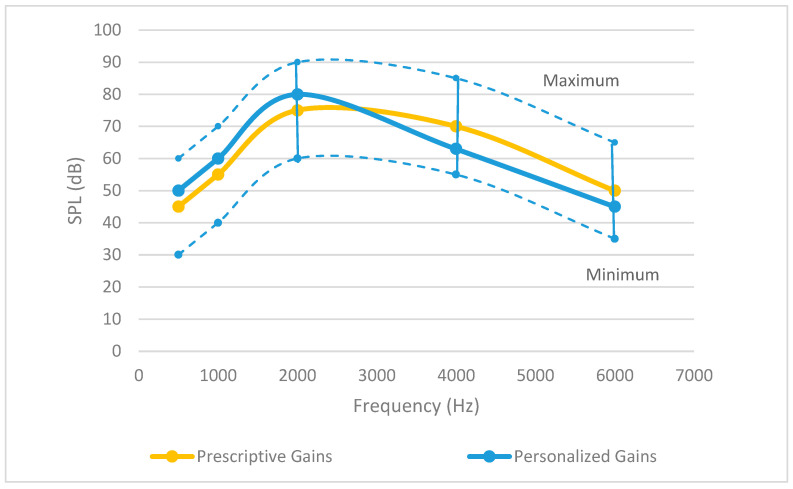
Prescription and personalized frequency–response curves indicating sound pressure level (SPL) versus audible frequencies. The range of personalized gains adjustment around prescribed gains across frequency bands is shown by the dashed lines (Maximum and Minimum).

Figure 3 illustrates how the prescription frequency–response curve is adjusted in a personalization process to obtain an individualized frequency–response curve. The range of personalized gains around prescribed gains includes the limits of loudness discomfort and hearing threshold. In [24], a review is presented covering various approaches for tailoring audio experiences. Three aspects of personalization were considered: spatial separation, the speech-to-noise ratio, and redundancy. It was reported that the speech-to-noise ratio played a dominant role, with spatial separation and redundancy playing comparatively lesser roles.

## 3. Methods: Personalization of Amplification in Hearing Aids

Previous works on the personalization of amplification in hearing aids can be categorized into two main groups according to their training procedures: (i) offline and (ii) online. In most studies, the paired comparison method was used to conduct training due to its simplicity and low cognitive load on users. In this section, the paired comparison method is presented first, followed by personalization studies categorized according to their offline and online training procedures. Then, the studies that have considered the effects of different audio environments on personalization are discussed.

### 3.1. Paired Comparison Method

Paired comparison has been a stalwart method used in hearing aid studies for nearly half a century [25]. The popularity of this method is due to its simplicity and the fact that it involves minimal auditory memory engagement [26]. While there are alternative methods such as round-robin tournaments, single-elimination tournaments, double-elimination tournaments, simple up–down procedures, and modified simplex procedures, these methods lack the inherent simplicity and straightforwardness of the paired comparison method.

As an example showing the utilization of paired comparisons in hearing aid studies, in [27], an investigation was conducted to compare two gain settings, labeled A and B. The laboratory environment in the study involved 20 participants, all with symmetrical hearing loss. This study incorporated two programs: a prescription made by a hearing aid manufacturer and an alternative prescription with a reduction in gain in a region around 1 kHz. Paired comparisons were used to examine attributes such as preference, speech intelligibility, comfort, and loudness for various sound stimuli played through loudspeakers. The results, derived from all the sound stimuli, revealed an equal preference for settings A and B. However, a more nuanced analysis showcased specific preferences: setting A was preferred for speech intelligibility, whereas setting B was favored for comfort. Notably, the participants preferred setting A in the context of soft speech and setting B in the context of cafeteria noise.

### 3.2. Categorization According to Training Procedure

#### 3.2.1. Offline Training

In this subsection, personalized hearing aid fitting studies are surveyed based on their offline training. In [28], multi-task learning was carried out based on hearing preferences. The goal was to leverage the similarities between the tasks. This study demonstrated the effectiveness of using a hierarchical semiparametric model based on Gaussian processes to achieve learning preferences. The experimental evaluation validated the preference learning approach using an audiological dataset. The experiments evaluated sound quality from normal-hearing and hearing-impaired participants through 576 pairwise comparisons. Preferences were collected based on the overall evaluation of the sound stimuli processed with different hearing aid settings. The objective was to assess whether the preferences of a new participant could be learned more accurately using the preferences of other participants. The hierarchical model used the Expectation Maximization (EM) algorithm [23] to gather data from a group of participants into a probability distribution serving as the prior for the test participant. The introduced model outperformed the two other examined models.

The study discussed in [29] formulated personalization as a linear regression problem. To address the challenges of low sample sizes and high feature numbers, the Bayesian approach of backfitting was employed to handle such features. The cited study compared this Bayesian approach to a heuristic feature selection approach. Synthetic data experiments with irrelevant and redundant features showed that the Bayesian backfitting approach achieved accuracies comparable to those of the heuristic approach for moderate sample sizes but required significantly longer training times. The authors then applied this Bayesian approach to hearing aid preference data to determine personalized features for hearing aid customization. Six individuals with normal hearing participated in a lab trial and were exposed to a stimulus combining speech and noise signals at various ratios. The participants adjusted the processing parameter while listening to a stimulus multiple times. The acoustic input and the processing parameters were stored to form input–output pairs for the offline training of a regression model. The results showed that four out of six participants indicated preferences based on two types of features.

The study reported in [30] addressed improving hearing aid fitting beyond the initial prescription-based fitting for individuals with hearing loss. It introduced a combination of a neural network and transfer learning to develop a fitting algorithm. The algorithm was trained offline on a dataset generated by hearing aid fitting softwares (NAL-NL1 and NAL-NL2). The effectiveness of the proposed fitting algorithm was assessed for three input sound levels (50 dB, 65 dB, and 80 dB) using the NAL-NL1 and NAL-NL2 prescriptions. The study provided performance comparisons across six different frequencies for each input sound level. The study also investigated the minimum number of fitting sessions required for effective training.

The study covered in [31] built upon the previous work by the same research group. It involved the evaluation of a neural-network-based personalized hearing-aid-fitting algorithm. The authors used 900 clinical samples for training and 78 for testing, with a dataset split ratio of 23 to 2. The dataset contained data on hearing loss at different frequencies as well as features such as age, sex, and ear type. The experimental results showed the performance of the algorithm for three input sound levels. It compared the proposed neural network fitting approach with and without the additional features. It was shown that the neural network algorithm with the additional features outperformed other approaches and was closely aligned with the original fitting sessions.

The study reported in [32] involved introducing a personalized compression approach based on deep reinforcement learning (DRL) that incorporates user feedback to optimize hearing aid settings based on individual preferences. In this approach, human feedback was used to model hearing preferences by considering a reward function that compared instances of two different compressed audio signals. Human preferences were collected through a hearing preference interface and a reward predictor that combined a convolutional neural network and a bidirectional long short-term memory network to conduct offline training based on the user’s feedback. This approach provided an active learning strategy for achieving personalized compression. In this study, two sets of experiments were conducted to assess the performance of the personalized DRL compression; the first set involved simulations of human-in-the-loop deep reinforcement learning, and the second set tested five participants with bilateral mild to moderate hearing loss. The DSLv5 prescriptive gains were mapped to five frequency bands to reduce computational complexity. The audiograms of the participants were acquired through a web-based hearing test forming the basis of the subsequent personalized compression training. A dataset of 210 pairs of sound files and human feedback was considered for this purpose. A comparative analysis was conducted between the personalized compression settings and the conventional or reference DSLv5 settings in paired comparisons of 60 sentences. The results demonstrated that participants with mild to moderate hearing loss preferred the personalized DSLv5 settings over the reference DSLv5 settings.

#### 3.2.2. Online Training

In this subsection, the studies that allowed online training for the personalization of hearing aid fitting are presented. Online training means that training can be conducted in an on-the-fly manner, and unlike offline training, it does not require the collection of data first. With regard to obtaining a practically deployable solution in the field or real-world audio environments, only online training approaches are of interest.

The study reported in [33] introduced an online personalization approach for setting compression ratios (CRs) in hearing aids. Initially, the hearing-aid-fitting process was conducted by setting gains across a number of frequency bands according to the DSLv5 prescription. This study focused on personalizing amplification by adjusting the CRs in each frequency band via the Maximum Likelihood Inverse Reinforcement Learning (MLIRL) machine learning technique. Paired comparisons of audio signals were carried out to gather preference feedback of an individual user, and then MLIRL was applied to find the most preferred combination of CRs across all the frequency bands for that user. The process involved defining a reward function through user feedback and updating it through paired comparison iterations. The developed MLIRL framework integrated user preferences in setting the optimum gain values across the frequency bands. This study reported the outcome for ten participants with mild to moderately severe hearing loss. The results showed a statistically significant preference for the personalized settings compared to the reference or conventional settings. Furthermore, a word recognition test was conducted, which showed the personalized settings did not compromise audibility or negatively impact word recognition in noisy conditions.

In [34], a study on Adaptive Dynamic Range Optimization (ADRO), it was demonstrated that the training could be conducted in an adaptive manner via the MLIRL method based on the incoming sound-level percentiles and a user’s hearing comfort level. The experimental setup involved ten participants with mild to moderately severe hearing loss. The results showed a statistically significant preference for the personalized ADRO over the standard ADRO. Additionally, the word recognition scores in noisy conditions revealed that there was no adverse impact of the personalized ADRO on speech comprehension.

The papers that follow do not explicitly clarify whether the training methodologies used were conducted in an offline or online manner. Nevertheless, it appears that the conceptual frameworks or mechanisms presented could be implemented in an online manner.

In [35], an interactive hearing aid personalization approach was introduced to achieve optimal individual settings through perceptual user feedback. This approach optimized the hearing aid settings or parameters by assuming that a user’s perception was encoded by an unobserved internal response function modeled by a non-parametric Bayesian regression model. The learning process was conducted in an iterative manner. Two studies, one involving two settings or parameters and the other involving four settings or parameters, were performed to demonstrate the personalization ability of this approach in a music scenario. This personalization work appeared in a patent addressing the topic of how to optimize the parameters of a hearing aid system [36].

A machine learning self-adjustment method was covered in [37]. This method allows users to optimize hearing aid settings based on personal preferences in various sound scenarios. In a lab study involving 20 participants with hearing loss, the method demonstrated a subjective benefit in audio quality. The study used a test protocol with two lab visits where participants adjusted hearing aid settings through a self-adjustment method named SSL (SoundSense Learn) in 12 different sound scenarios categorized by the three attributes of audio quality, listening comfort, and speech clarity. The results showed a significant improvement in audio quality with SSL adjustments. However, listening comfort improvements varied across scenarios, and no significant improvement was observed in speech clarity. This study, again, highlighted the need for an individualized approach to creating and optimizing hearing aid fittings.

A similar study discussed in [38] explored a machine learning approach for adjusting hearing aid gain settings based on user preferences obtained through an iterative paired comparison procedure. This study used 20 hearing-impaired participants who underwent hearing aid adjustments using the three settings of REF (Reference), UNI (Universal), and SSL. Twelve sound scenarios were used, and the participants rated each setting in terms of audio quality, listening comfort, and speech clarity. The SSL setting involved adjustments based on the SSL technology. The results revealed notable improvements in audio quality. Individual participants showed diverse preferences, emphasizing the subjective nature of hearing aid experiences. Overall, this study highlighted the importance of individualized hearing aid settings and the potential benefits of SSL technology.

The authors of the study reported in [39] used Bayesian learning to optimize sound settings in hearing aids. SSL technology was utilized to personalize sound signals based on user preferences gathered via A–B comparisons in a smartphone app. This study highlighted how SSL led to improved sound quality in various listening environments. The significance of the SSL data in enhancing hearing was emphasized in this study.

In [40], over-the-counter hearing aids, intended to make hearing healthcare more affordable and accessible, were evaluated by allowing end-users to configure such hearing aids themselves. An approach involving the division of a 24-dimensional configuration space into presets was considered to meet the needs of a significant portion of individuals with mild-to-moderate hearing loss. An online agent then learned the best preset through a series of pairwise comparisons. This study identified a relationship between user preferences and presets, leading to the development of a Two-Phase Personalizing (TPP) algorithm that refined an existing dueling multi-armed bandit (MAB) algorithm. The 24-dimensional configuration space was divided into 15 presets based on audiograms from the National Health and Nutrition Examination Survey (NHANES). The Borda score was used to indicate preferences, and the users typically had one or a few highly preferred presets. It was shown that TPP outperformed two other algorithms (Active Ranking and SAVAGE) in simulations.

The study reported in [41] explored a self-fitting interface for fitting hearing aids by allowing users to choose their own signal-processing parameters through a two-wheel interface. In a month-long field trial, the participants with hearing loss either self-selected parameters using the new interface or relied on the parameters that were set by a clinician. The participants in the self-group selected gain settings that correlated with the severity of hearing loss. The self-selected gains were slightly lower than the audiologist-selected gains. The audiologist-selected gains had a slightly steeper correlation with hearing loss severity than the self-selected gains. Essentially, the participants in the self-group reported higher satisfaction ratings than those in the other group. Both groups preferred their self-selected settings over the clinical fit, with the self-group showing a stronger preference. It is important to note, however, that users of hearing aids often prefer initial settings that are lower than optimal, but their tolerance of amplification changes over time as they adjust. Thus, users may underfit themselves without guidance from a clinician, underscoring a major limitation in the self-fit approach.

### 3.3. Overview Comparison of Personalization Studies

The methodology employed in the training process is crucial in hearing aid personalization. Key factors such as the online nature of training, real-time implementation, and subject testing are often sought after. If the methodology is tested on participants, it essentially establishes the usability of the personalization process. The distinction between laboratory audio environment studies and those conducted in real-world audio environments is also pivotal. Generally speaking, settings customized in a laboratory audio environment may not work well when users are situated in real-world audio environments. Hence, when a study tests its methodology in a real-world audio environment, the robustness of the study is examined in terms of individual preferences in different audio environments. Table 1 provides an overview comparison of whether the training occurred online (or even if that is a possibility), whether a real-time implementation was conducted, in what audio environment the study was conducted, the machine learning methodology used (if used at all), the source of data, personalized variables, and the personalization approach studied.

### 3.4. Studies Related to Environmental Context

In the context of personalizing hearing aid algorithms, it is necessary to devise solutions that seamlessly adapt to diverse audio environments. This section delves into works that have taken into consideration environmental settings in different ways or have considered the daily routines of hearing aid users.

The study reported in [42] introduced a real-time unsupervised background noise classification algorithm to identify different types of background noise without prior training. The algorithm modified the OFC (Online Frame-based Clustering) algorithm and included feature extraction, a fading function, and classification smoothing. Feature vectors were extracted from captured signal frames using band periodicity and band entropy attributes. A framing step was used to buffer input samples of features, and a clustering decision was made using the information in a frame. A fading function and classification smoothing were incorporated to improve performance. Experiments were conducted to evaluate the impact of the parameters of chunk size and segment length on the outcome. Field testing demonstrated real-time performance in different noise environments, and the algorithm successfully adapted to changing noise conditions. This work introduced a real-time unsupervised background noise classification algorithm that holds promise for enhancing the adaptability of hearing aids to changing audio environments.

The study covered in [43] developed a model for personalizing hearing aids by tuning parameters that adapted to environmental conditions. It involved using a control wheel (CW) for adjustments made by users and a learning process using explicit consent moments. The model incorporated an environment coder (EVC) to extract features from the input signal, enabling the learning of environmental conditions. The goal was to maximize user satisfaction by updating tuning parameters based on consent moments. It included both the offline and online training of a probabilistic generative model. This approach was evaluated in a simulated learning volume control scenario, which showed the potential benefit of online learning for personalized hearing aid adjustment. In a field trial, the participants used an experimental hearing instrument for six weeks, adjusting it to improve their listening experiences. The examination of the participants’ preferences revealed a non-linear relationship between noise reduction and signal-to-noise ratios (SNRs) and power levels.

Notably, a noise reduction of about 1 dB was observed for high power and a high SNR, whereas nearly 7 dB was observed for low power and a low SNR. Most participants exhibited symmetric noise reduction preferences, but some noted asymmetry, underscoring the need for personalization.

Internet-connected hearing aids allow the utilization of smartphone apps, permitting users to set optimal settings based on the surrounding audio environment. The study reported in [44] involved seven participants with binaural hearing loss using a commercial hearing aid connected to iPhones with a custom app. The participants participated a four-week experiment to optimize three audiological parameters (noise reduction and directionality, brightness, and soft gain). Participants used a smartphone app to compare parameter levels in various real-world situations, reporting preferences, environmental context, motion state, audiological intent, and perceived usefulness. In the final week, the participants blindly compared a personalized configuration based on their most frequently selected preferences to a configuration determined through a standard clinical workflow. The results showed diverse audiological preferences among the participants. Brightness was consistently perceived as the most useful parameter, whereas noise reduction and directionality were perceived as the least useful for five out of seven participants.

In [45], the authors introduced a Daily Routine Recognition (DRR) system that processed inputs from Audio and Acceleration (ACC) sensors to recognize routine behaviors and environments. The approach involved building feature representations, applying supervised learning with various classifiers and evaluating their performances. The features were derived from raw acceleration and precomputed audio features, and these were used to distinguish classes representing routine behavior and environments. The features of the ACC sensors included statistical measures such as mean, axes correlation, variance, and mean crossing rate. The audio features included voice activation, auto-correlation of samples, wind activity, maximum level, spectral centroid, and speech characteristics. The processing scheme included concatenation, statistical evaluation, feature extraction, and selection. The classifiers used for offline and online learning included a deep neural network (DNN), random forest (RF), multi-layer perceptron (MLP), k-nearest neighbor (kNN), Gaussian mixture model (GMM), Naïve Bayes (NB), and support vector machine (SVM). The evaluation was conducted using cross-validation schemes for offline classification and online simulation to assess performance improvement with daily updates. The results indicated that the DNN, MLP, and RF classifiers performed well for offline classification, with the DNN and MLP classifiers significantly outperforming the other classifiers. In online simulations, the MLP outperformed the other classifiers, and online updates improved this performance.

In [46], daily routines were recognized using a sequence-learning model that involved feature representation and using a Long Short-Term Memory (LSTM) network together with a Hidden Markov Model (HMM) for sequence behavior learning. The features were derived from two accelerometer (ACC) sensors and audio data, and a statistical representation was developed on both activity primitive and routine levels. A sliding window was used to extract features such as mean, axes correlation, variance, and mean crossing rate. The study evaluated the performance of different sequence learners using a feature selection algorithm. The evaluation included non-sequence learners (RF, MLP, and GMM) and sequence learners (HMM and LSTM). The results obtained indicated that sequence learning, especially when combined with RF and MLP learning, improved classification performance. The models were compared based on accuracy, F1-score, and confusion matrix. The findings indicated the effectiveness of sequence learning in recognizing daily routines.

The study reported in [47] addressed the user experiences of deaf or hard-of-hearing (DHH) individuals when recording sound samples for a personalized sound recognition system. It involved 14 DHH participants in a three-part study: an initial interview, a week-long field study of recording samples, and a follow-up interview with a design probe activity. The participants provided information on their demographics, hearing loss, relationship to sound, and familiarity with machine learning. The study included the use of a Teachable Machine for sound classification, a field study using the Rev Recorder app, and a final interview with a focus on user experience and design ideas for improving sound recognition tools. The results revealed the challenges in recording diverse and nuanced sound classes, the participants’ considerations for decision boundaries and sampling difficulty, and the importance of feedback during and after the recording process.

Combining the data from hearing aids with the data from other sensors, such as those measuring heart rate, motion, and location, further insight into the context of the sounds hearing aid users detect in real-world audio environments can be gained. In other words, gathering information from other sensors would make personalizing hearing aid settings more effective.

The current usage of modern hearing aids is hindered by a perceived lack of benefit, stemming from limitations in the fitting procedure. This procedure often overlooks two crucial factors: (1) perceptual differences among users not solely explained by audiometric results, and (2) variations in individual context-specific preferences. The advent of smartphone-connected hearing aids presents an opportunity to address these issues by enabling a dynamic adaptation of settings based on users’ changing needs. The work in [48] explored the potential of modeling user auditory intent through context collected via mobile devices. It discussed the relevance of various types of contextual information for learning the situation-specific intent and preferences of hearing-impaired users. This work also provided real-life examples to illustrate these concepts.

The work in [49] focused on evaluating the ability of a Gaussian Process (GP) model to learn the preferences of users using Bayesian optimization in a simulation environment. The simulation compared the performance of the model with an oracle mean model. The results showed that the GP model outperformed the oracle mean model. The discussion emphasized the potential of using simulations for studying adaptive personalization systems and suggested that simulations could guide the development of more accurate models in real-world applications.

The study reported in [50] utilized data from a large database reflecting the hearing aid (HA) usage of individuals who subscribed to a hearing fitness feature through a hearing aid manufacturer smartphone app. The participants were users of =those hearing aid who used this feature for at least ten days. The data underwent pre-processing to estimate hourly HA use, address temporary disconnections, and remove days with atypical patterns. The analysis involved exploring the amount of HA use and the number of clustering users based on their HA use patterns. This study analyzed 453,612 days of HA use corresponding to 15,905 users. On average, HAs were used for 10.55 h per day, with significant variability. Three user groups (full-day users, afternoon users, and sporadic evening users) were identified based on their HA use patterns.

The studies reviewed in this section highlight the diverse ways researchers are addressing the crucial role of environmental context in enhancing hearing aid functionality. Table 2 provides an overview or summary of these ways.

## 4. Research Challenges and Future Directions

Addressing the challenges associated with real-world audio environments requires a multifaceted approach. The dynamic and diverse nature of these environments poses a significant hurdle, demanding the implementation of personalization solutions that can adapt seamlessly to different settings, noise levels, and acoustic conditions. Moreover, the diversity in user preferences and hearing profiles adds another layer of complexity. Designing a personalization solution capable of effectively learning and adapting to these individual differences is a major challenge. Creating user-friendly interfaces on smartphones is essential for seamless interaction with users providing feedback and preferences effortlessly. Additionally, the adaptability of a personalization solution and the potential learning curve for users in understanding and utilizing the personalization capability present further challenges.

It is worth noting that although the focus of this review has been on hearing aids, the concept of personalizing or individualizing amplification can be applied to other hearing-assistive devices such as cochlear implants, personal sound amplifiers, and audio-processing devices. Moving forward, researchers working with other hearing-assistive devices can consider implementing similar approaches to those covered in this paper in order to study the applicability of personalization across other platforms.

Looking towards the future, the evolution of adaptive machine learning algorithms holds promise in addressing these challenges. Implementing algorithms that can learn and adapt in real-time based on user preferences will significantly enhance personalization. Context-aware personalization is another future direction, in which solutions that automatically identify a user’s operating audio environment and adjust settings accordingly are being explored. Introducing effective feedback mechanisms, such as surveys and continuous feedback loops, will be useful for refining personalization solutions based on a user’s experiences. Integration with wearable devices is also a potential avenue for gathering additional contextual data with which to further enhance personalization.

A user-centric design approach is imperative to ensure effectiveness and user acceptance. Involving users in the design process, conducting usability studies, and iteratively refining the user interface will lead to the creation of intuitive and user-friendly personalization solutions. Standardization and interoperability efforts are also necessary to enable seamless integration between different hearing aid devices and smartphone platforms, offering users a consistent experience across devices. Rigorous clinical validation through trials conducted in various real-world scenarios will build confidence in a real-time personalization technology among users, particularly for over-the-counter hearing aids, by empowering them to adjust settings according to their preferences. Overall, the future of personalized hearing aids involves a convergence of technological advancements, user-centric design principles, and robust validation processes to overcome existing challenges and enhance user experience.

## Figures and Tables

**Table 1:** Overview comparison of machine learning-based personalization approaches for hearing aid amplification.

Author, Paper No. (Year)	Training	Real-Time Implementation	Data Source	Audio Environment	Personalized Variable	Machine Learning Methodology	Approach
Birlutiu et al. [28] (2010)	Offline	no	Pairwise comparison feedback	dataset	Hearing aid parameter settings	Hierarchical Preference Learning and Expectation Maximization	Learning from similar tasks with multiple participants
Ypma et al. [29] (2007)	Offline	yes	Synthesized data and human test results	dataset and lab	Hearing aid parameter settings	Linear Regression and Bayesian Learning	Formulating hearing aid personalization as a linear regression problem
Mondol et al. [30] (2019)	Offline	no	Prescription fitting results from 1100 participants	dataset	Insertion gain	Neural Network (NN) with Transfer Learning	Predicting insertion gain based on hearing loss characteristics and input levels
Mondol et al. [31] (2022)	Offline	no	Clinical fitting data	dataset	Insertion gain	NN with Transfer Learning	Taking age, sex, and ear type into consideration
Alamdari et al. [32] (2020)	Offline	yes	Pairwise comparison feedback	lab	Compression ratio	Deep Reinforcement Learning	Providing human-in-loop deep reinforcement learning
Akbarzadeh et al. [33] (2022)	Online	yes	Pairwise comparison feedback	lab	Compression ratio	Maximum Likelihood Inverse Reinforcement Learning	Online machine learning personalization
Ni et al. [34] (2022)	Online	yes	Pairwise comparison feedback	lab	Comfort target	Maximum Likelihood Inverse Reinforcement Learning	Personalization of an adaptive prescription
Nielsen et al. [35] (2014)	Possibility for online exists	yes	Pairwise comparison feedback	lab	Gain set	Gaussian Processes (GPs) and Active Learning	Obtaining an individualized setting from direct perceptual user feedback
Jensen et al. [37] (2019)	Possibility for online exists	yes	Pairwise comparison feedback	lab	Hearing aid parameter settings	Study of a commercialized learning algorithm in [35]	Performing personalization in 12 sound scenarios
Søgaard et al. [38] (2019)	Possibility for online exists	yes	Pairwise comparison feedback	lab	Gain set	Study of a commercialized learning algorithm in [35]	Adjusting gains based on user preference in an iterative paired-comparison manner
Balling et al. [39] (2021)	Possibility for online exists	yes	Pairwise comparison feedback	lab and real-world	Sound settings	Study of a commercialized learning algorithm in [35]	Describing a mechanism that operates continuously on user inputs
Vyas et al. [40] (2022)	Possibility for online exists	yes	Pairwise comparison feedback	dataset and lab	Gain set	Dueling Multi-Armed Bandit (MAB) Learning	Reducing the number of comparisons to identify a user’s preferred preset
Sabin et al. [41] (2020)	Possibility for online exists	yes	Pairwise comparison feedback	lab and real-world	Gain set	Not mentioned explicitly, a self-fitting interface	Allowing users to have simultaneous control of gain and compression in each frequency band

**Table 2:** Environmental context in hearing aid personalization studies.

Author, Paper No. (Year)	Environmental Context Studied
Saki et al. [42] (2017)	Classification of environmental noise signals
Ypma et al. [43] (2008)	Personalization of tuning parameters with a focus on noise reduction
Pasta et al. [44] (2019)	Modeling the surrounding environment and recommending optimal settings in different contexts
Kuebert et al. [45] (2021)	Daily routine recognition for personalizing hearing aid configurations
Kuebert et al. [46] (2021)	Enhancing daily routine recognition using sequence learning techniques
Korzepa et al. [48] (2018)	Representation of user intentions related to hearing preferences with context gathered through mobile devices
Korzepa et al. [49] (2020)	Creating a simulation-based framework for online contextual personalization of hearing aids
Pasta et al. [50] (2021)	Ascertaining daily patterns and variability in hearing aid usage to gain insights into user behavior

## Data Availability

Data are contained within the article.

## References

[1] Dell’Antônia S.F., Ikino C.M.Y., Filho W.C. (2013). Degree of satisfaction of patients fitted with hearing aids at a high complexity service. Braz. J. Otorhinolaryngol..

[2] Hearing and Quality of Life in Older Adults. https://ahassavannah.com/hearing-and-quality-of-life-in-older-adults/.

[3] Dalton D.S., Cruickshanks K.J., Klein B.E.K., Klein R., Wiley T.L., Nondahl D.M. (2003). The impact of hearing loss on quality of life in older adults. Gerontologist.

[4] Thomson R.S., Auduong P., Miller A.T., Gurgel R.K. (2017). Hearing loss as a risk factor for dementia: A systematic review. Laryngoscope Investig. Otolaryngol..

[5] Banerjee S. (2011). The Compression Handbook.

[6] What Is an Audiogram?—Understanding Hearing Test Results. https://www.babyhearing.org/what-is-an-audiogram.

[7] Vogel A.D., McCarthy P.A., Bratt G.W., Brewer C. (2007). The clinical audiogram. Commun. Disord. Rev..

[8] Hearing Aids: Uses & How They Work. https://my.clevelandclinic.org/health/treatments/24756-hearing-aids.

[9] What Is a Hearing Aid Prescription?. https://hearingup.com/videos/what-is-a-hearing-aid-prescription.

[10] Venema T. The NAL-NL1 Fitting Method. https://www.audiologyonline.com/articles/the-nal-nl1-fitting-method-1260.

[11] Keidser G., Dillon H., Carter L., O’Brien A. (2012). NAL-NL2 empirical adjustments. Trends Amplif..

[12] Keidser G., Dillon H., Flax M., Ching T., Brewer S. (2011). The NAL-NL2 prescription procedure. Audiol. Res..

[13] Polonenko M.J., Scollie S.D., Moodie S., Seewald R.C., Laurnagaray D., Shantz J., Richards A. (2010). Fit to targets, preferred listening levels, and self-reported outcomes for the DSL v5 hearing aid prescription for adults. Int. J. Audiol..

[14] (2023). DSL^®^ v5 by Hand. https://www.dslio.com/wp-content/uploads/2014/06/DSL-5-by-Hand.pdf.

[15] Bagatto M., Moodie S., Scollie S., Seewald R., Moodie S., Pumford J., Liu K.P.R. (2005). Clinical protocols for hearing instrument fitting in the desired sensation level method. Trends Amplif..

[16] Blamey P.J. (2005). Adaptive dynamic range optimization (ADRO): A digital amplification strategy for hearing aids and cochlear implants. Trends Amplif..

[17] Blamey P., James C., Wildi K., McDermott H., Martin L. (2004). Adaptive Dynamic Range of Optimization Sound Processor. U.S. Patent.

[18] Blamey P., James C., McDermott H., Martin L., Wildi K. (2008). Adaptive Dynamic Range Optimization Sound Processor. U.S. Patent.

[19] Blamey P., James C., McDermott H., Martin L., Wildi K. (2011). Adaptive Dynamic Range Optimization Sound Processor. U.S. Patent.

[20] Plomp R. (1994). Noise, Amplification, and Compression: Considerations of Three Main Issues in Hearing Aid Design. Ear Hear..

[21] Hickson L.M.H. (1994). Compression Amplification in Hearing Aids. Am. J. Audiol..

[22] Lybarger S.F. (1978). Selective Amplification—A Review and Evaluation. Ear Hear..

[23] Johansen B., Petersen M.K., Korzepa M.J., Larsen J., Pontoppidan N.H., Larsen J.E. (2017). Personalizing the Fitting of Hearing Aids by Learning Contextual Preferences from Internet of Things Data. Computers.

[24] Ward L., Shirley B.G. (2019). Personalization in object-based audio for accessibility: A review of advancements for hearing impaired listeners. J. Audio Eng. Soc..

[25] Amlani A.M., Schafer E.C. (2009). Application of paired-comparison methods to hearing aids. Trends Amplif..

[26] Kuk F.K. (2002). Paired comparisons as a fine-tuning tool in hearing aid fittings, strategies for selecting and verifying hearing aid fittings. Strateg. Sel. Verif. Hear. Aid Fitt..

[27] Dahlquist M., Larsson J., Hertzman S., Wolters F., Smeds K. Predicting individual hearing-aid preference in the field using laboratory paired comparisons. Proceedings of the International Symposium on Auditory and Audiological Research.

[28] Birlutiu A., Groot P., Heskes T. (2010). Multi-task preference learning with an application to hearing aid personalization. Neurocomputing.

[29] Ypma A., Ozer S., van der Werf E., de Vries B. Bayesian Feature Selection for Hearing Aid Personalization. Proceedings of the IEEE Workshop on Machine Learning for Signal Processing.

[30] Mondol S.R., Lee S. (2019). A Machine Learning Approach to Fitting Prescription for Hearing Aids. Electronics.

[31] Mondol S., Kim H.J., Kim K.S., Lee S. (2022). Machine learning-based hearing aid fitting personalization using clinical fitting data. J. Healthc. Eng..

[32] Alamdari N., Lobarinas E., Kehtarnavaz N. (2020). Personalization of Hearing Aid Compression by Human-in-the-Loop Deep Reinforcement Learning. IEEE Access.

[33] Akbarzadeh S., Lobarinas E., Kehtarnavaz N. (2022). Online Personalization of Compression in Hearing Aids via Maximum Likelihood Inverse Reinforcement Learning. IEEE Access.

[34] Ni A., Akbarzadeh S., Lobarinas E., Kehtarnavaz N. (2022). Personalization of hearing aid fitting based on adaptive dynamic range optimization. Sensors.

[35] Nielsen J.B.B., Nielsen J., Larsen J. (2014). Perception-based personalization of hearing aids using Gaussian processes and active learning. IEEE/ACM Trans. Audio Speech Lang. Process..

[36] Nielsen J.B.B., Ougaard A., Molgaard L.L., Aleksander C., Jespersen B. (2023). Method of Optimizing Parameters in a Hearing Aid System. U.S. Patent.

[37] Jensen N.S., Balling L.W., Nielsen J.B.B. Effects of Personalizing Hearing-Aid Parameter Settings Using a Real-Time Machine-Learning Approach. Proceedings of the 23rd International Congress on Acoustics.

[38] Jensen N.S., Hau O., Nielsen J.B.B., Nielsen T.B., Legarth S.V. (2019). Perceptual Effects of Adjusting Hearing-Aid Gain by Means of a Machine Learning Approach Based on Individual User Preference. Trends Hear..

[39] Balling L.W., Mølgaard L.L., Townend O., Nielsen J.B.B. The Collaboration between Hearing Aid Users and Artificial Intelligence to Optimize Sound. Proceedings of the Seminars in Hearing.

[40] Vyas D., Brummet R., Anwar Y., Jensen J., Jorgensen E., Wu Y.-H., Chipara O. (2022). Personalizing over-the-counter hearing aids using pairwise comparisons. Smart Health.

[41] Sabin A.T., Van Tasell D.J., Rabinowitz B., Dhar S. (2020). Validation of a Self-Fitting Method for Over-the-Counter Hearing Aids. Trends Hear..

[42] Saki F., Kehtarnavaz N. (2017). Real-time unsupervised classification of environmental noise signals. IEEE/ACM Trans. Audio Speech Lang. Process..

[43] Ypma A., Geurts J., Özer S., Van der Werf E., De Vries B. (2008). Online personalization of hearing instruments. EURASIP J. Audio Speech Music Process..

[44] Pasta A., Petersen M.K., Jensen K.J., Larsen J.E. Rethinking Hearing Aids as Recommender Systems. Proceedings of the CEUR Workshop.

[45] Kuebert T., Puder H., Koeppl H. (2021). Daily Routine Recognition for Hearing Aid Personalization. SN Comput. Sci..

[46] Kuebert T., Puder H., Koeppl H. (2021). Improving Daily Routine Recognition in Hearing Aids Using Sequence Learning. IEEE Access.

[47] Goodman S.M., Liu P., Jain D., McDonnell E.J., Froehlich J.E., Findlater L. (2021). Toward User-Driven Sound Recognizer Personalization with People Who Are d/deaf or hard of hearing. Proc. ACM Interact. Mob. Wearable Ubiquitous Technol..

[48] Korzepa M.J., Johansen B., Petersen M.K., Larsen J., Larsen J.E., Pontoppidan N.H. Modeling User Intents as Context in Smartphone-Connected Hearing Aids. Proceedings of the Adjunct Publication of the 26th Conference on User Modeling, Adaptation and Personalization.

[49] Korzepa M., Petersen M.K., Larsen J.E., Mørup M. Simulation Environment for Guiding the Design of Contextual Personalization Systems in the Context of Hearing Aids. Proceedings of the 28th ACM Conference on User Modeling, Adaptation and Personalization.

[50] Pasta A., Szatmari T.-I., Christensen J.H., Jensen K.J., Pontoppidan N.H., Sun K., Larsen J.E. (2021). Clustering users based on hearing aid use: An exploratory analysis of real-world data. Front. Digit. Health.

